# BLOOD URINE POSITIVITY RATE DIPSTICK METHOD ON THE INCIDENCE OF ANEMIA IN URINARY TRACT INFECTION PATIENTS

**DOI:** 10.21010/Ajidv19i2.2

**Published:** 2025-04-07

**Authors:** Siti Zaetun, Lalu Srigede

**Affiliations:** 1 Department of Medical Laboratory Technology, Politeknik Kesehatan Mataram, Mataram, Indonesia; 2 Department of Medical Laboratory Technology, Politeknik Kesehatan Mataram, Mataram, Indonesia

**Keywords:** Blood, Urine, Dipstick method, Anemia, and Hemoglobin

## Abstract

**Background:**

The prevalence of anemia in children suffering from urinary tract infections with positive hematuria is very high. UTI is confirmed by a complete urine examination including macroscopic, chemical and microscopic examinations. Chemical examination can be carried out using the dipstick method, while urine microscopy involves looking at the image of the urine sediment. One of the parameters on the dipstick is blood. Examination with a reagent strip (dipstick) function to detect erythrocytes, free hemoglobin and myoglobin. So, the presence of erythrocytes, myoglobin or hemoglobin in the urine will give a positive result on the urine blood dipstick parameters, which means hematuria occurs.

**Materials and Methods:**

This is a cross-sectional analytical observational method, namely by comparing the results of blood urine examination using the dipstick method, with the results of the erythrocyte index and hemoglobin levels on the incidence of anemia in UTI patients.

**Results:**

The results of the blood urine dipstick examination obtained the highest positive value of 3. The average values for Hb, MCV, MCH, MCHC were respectively 10.9 g/dL, 83.3 fL, 28.5 pg, and 34.24%.

**Conclusion:**

The results of the Pearson correlation test obtained a degree of relationship of -0.363 which indicates a weak correlation. Blood urine is negatively related to the incidence of anemia (Hb levels), so that the higher the positive blood urine value, the lower the Hb value.

## Introduction

Urinary tract infection (UTI) is an infection due to the entry of pathogenic microorganisms in the kidneys, ureters, bladder, or urethra, causing inflammation and bacteriuria (Inayati and Falah, 2014). UTI is the second most infectious disease in the world community after respiratory tract infections (Zhou *et al.*, 2023). In Indonesia, based on data from the Ministry of Health of the Republic of Indonesia in 2014, the prevalence of UTI has reached 90-100 cases per 100,000 population per year or around 180,000 new cases per year (Vidiasari, 2016).

Most UTI cases are caused by: *Enterobacteriaceae* most important types are *Escherichia coli, Klebsiella* and *Species Enterobacter* (Sugianto *et al.*, 2020). Infectious diseases by microorganisms are one of the important factors related to the high prevalence of anemia, especially in women and children (Chaparro and Suchdev, 2019). Anemia is a condition of decreased hemoglobin, hematocrit, and red blood cell counts below normal values (Kurniawan, 2018).

In addition, microorganisms are also one of the sources of infection in sepsis patients, there was a correlation between sepsis patients and anemia with *Acinetobacter baumannii* (26.08%) as the common microorganism found (Hatman, Semedi and Budiono, 2021) . In this case, microorganisms are identified in 60–65% of sepsis patients and 75% of sepsis-getting patients in the ICU. Among the most important causes of anemia are sepsis, excessive blood loss, lack of erythropoietin production and iron deficiency related to the immune system (Vincent, 2002). Research by (Chaparro and Suchdev, 2019) also obtained the results of a high incidence of anemia in children suffering from UTIs and a high prevalence of anemia in children suffering from urinary tract infections with positive hematuria.

UTI diagnosis done with complete urine examination includes macroscopic, chemical, and microscopic examinations. Chemical examination can be done by dipping method (dipstick), whereas microscopic examination of the urine by looking at the image of sediments in the urine (Rinawati and Aulia, 2022). One of the parameters on the dipstick is blood. Examination with reagent strips (dipstick) serves to detect erythrocytes, free hemoglobin, and myoglobin (Riswanto;Rizki, 2015). So that the presence of erythrocytes, myoglobin and hemoglobin in the urine will give a positive result on the urine blood dipstick parameter which means hematuria occurs.

Hematuria is an abnormal condition characterized by the presence of erythrocytes in the urine. Damage in the structure of the urinary tract causes hematuria so that blood will mix with urine in an abnormal state. Erythrocytes are normally absent in the urine, although their presence is 1–2 per large field of view is still considered as such (Dwiyana and Astrawinata, 2016).

## Materials and Methods

### Study Design

The study was conducted by comparing the results of the dipstick method in the blood urine examination, against the results of erythrocyte index and hemoglobin levels against the incidence of anemia in UTI patients. The duration of the study was four months (January, 2023 to April, 2023).

### Samples

The population of this study was all Urinary Tract Infection patients at RSUD Kota Mataram. While the sample used are those of patients with Urinary Tract Infection (UTI) with positive blood urine results.

### Ethical Approval

This study was approved by the Medical and Health Research Ethics Committee of Politeknik Kesehatan Mataram, with approval number: LB.01.03/6/108/2023, dated 15 March 2023.

### Informed Consent

Informed consent was obtained from all individual participants included in this study.

### Data Collection 

The sampling techniques used in this study were *non-probability sampling* by method of *accidental sampling* (Sugiyono, 2017).

### Laboratory Methods

### Blood Urine Test

Examination with reagent strips (dipstick) detects erythrocytes, free hemoglobin, and myoglobin, but the reaction is more sensitive to hemoglobin and myoglobin than erythrocytes. The reagent pad is impregnated with tetramethylbenzidine chromogen and peroxide. The dipstick procedure is based on the peroxidase activity of hemoglobin and myoglobin catalyzing the oxidation of chromogens with hydrogen peroxide.

Erythrocytes in the urine will be lysed on the test pad in the presence of peroxidase activity, then free hemoglobin will act with reagents and will produce green dots on a yellow or orange background. Thus, the presence of intact erythrocytes will give a reaction in the form of green spots, while free hemoglobin and myoglobin will produce a green color what may range to dark blue. The absence of hemolysis can indicate a negative result even if erythrocytes are found in the sediment (Riswanto;Rizki, 2015).

### Anemia Diagnosis

Diagnosis of anemia is carried out by hematological and non-hematological laboratory examinations (Handayani, Wiwik; Haribowo, 2008).
1. Hematological laboratory examination
Hematological laboratory examination can be carried out gradually
a) Filter tests are performed in the early stages of each case of anemia. Filter tests include checking hemoglobin levels, erythrocyte indices (MCV, MCH, and MCHC), and peripheral blood smears.
b) Routine examination is an examination to determine abnormalities in the leukocyte and platelet system, including checking the sedimentation rate of blood, differential count, and reticulocyte count.
c) Bone marrow examination is done to get a definitive diagnosis, but there are some cases that do not require this examination.
d) Examination of special indications is an examination carried out if you have a suspected initial diagnosis so that its function is to confirm the suspected diagnosis.
(1) Iron deficiency anemia: examination of serum iron, TIBC, transferrin saturation, and serum ferritin.
(2) Megaloblastic anemia: examination of blood folic acid, vitamin B12.
(3) Hemolytic anemia: reticulocyte count, combs test, and Hb electrophoresis.
(4) Anemia in acute leukemia is usually carried out cytochemical examination
1) Non-hematological laboratory examination
a) Renal failure examination
b) Endocrine examination
c) Uric acid examination
d) Liver function test
e) Bacterial culture
2) Other supporting checks
In some cases of anemia, supporting examinations are also needed among others:
a) Biopsy of the gland followed by histopathological examination.
b) Radiology: thoracic, bone survey, ultrasound, or lymphangiographic.
c) Molecular Biology Examination (PCR, FISH).

### UTI Diagnose

UTI diagnosis is made based on the presence of symptoms, or clinical manifestation and laboratory examination of urine (Andriani, 2019). Urinary tract infection can be identified by several symptoms such as fever, difficulty urinating, pain after defecation (terminal dysuria), frequent urination, sometimes feeling hot when urinating, low back pain and suprapubic pain.

Establishing the diagnosis of UTI in addition to clinical manifestations also requires supporting examinations, namely laboratory examinations. Laboratory tests for UTIs consist of urinalysis and urine culture. A complete urinalysis consists of macroscopic, chemical, and microscopic examinations. Macroscopic examination includes description of volume, color, clarity, odor, and specific gravity. Chemical inspection can be carried out using commercial reagent strips (dipsticks). Chemical tests associated with UTIs themselves include pH, leukocyte esterase, and nitrite. Meanwhile, microscopic examination includes the presence of leukocytes, erythrocytes, and bacteria(Rinawati and Aulia, 2022).

## Result

**Figure 4.1:**
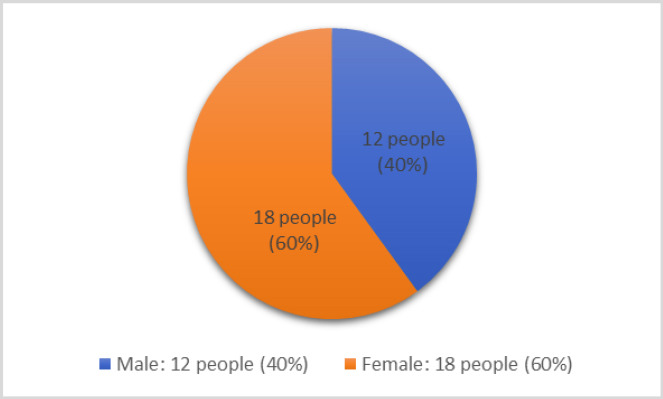
number of samples by sex

**Figure 4.2:**
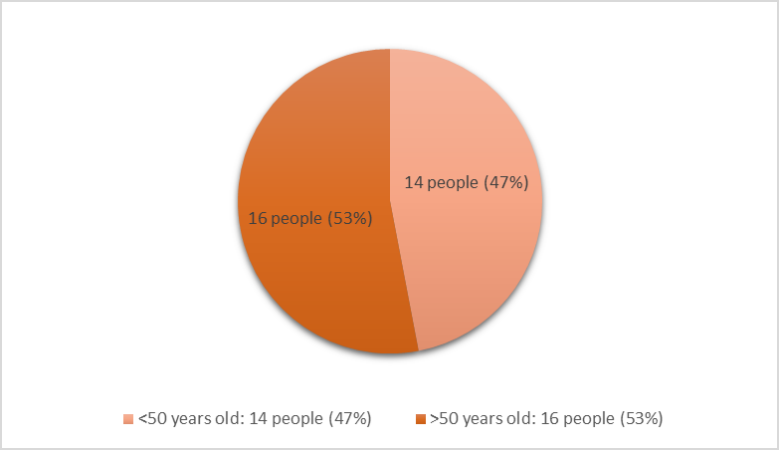
number of samples based on age of respondents

Characteristics of UTI patient respondents by sex in figure 4.1 below:

Based on figure 4.1, the sample of UTI patients in women is more than men, namely women 18 people (60%) while men 12 people (40%) from a total of 30 study samples. The characteristics of UTI patient respondents based on age in figure 4.2 below:

Based on figure 4.2, the sample of UTI patients with an age range over 50 years is more than 16 people (53%), compared to the age of less than 50 years, a total of 14 people (47%) from a total of 30 study samples.

The results of dipstick method blood urine examination and erythrocyte index (Hb, MCV, MCH, MCHC) in UTI patients are complete in table 4.1 below:

**Table 4.1:** Results of blood urine and erythrocyte index in UTI patients

Sample Code	Gender	Age (Years)	Examination Results				
			Blood Urine	Hb (g/dL)	Erythrocytes Index		
					MCV (fl)	MCH (pg)	MCHC (%)
01	P	28	+1	10.6	73.1	24.0	33.1
02	P	39	+2	11.1	85.1	29.1	34.2
03	P	41	+1	10.3	81.5	28.5	35.0
04	P	5	+3	9.4	71.1	23.8	33.5
05	L	75	+3	10.5	80.3	28.4	35.4
06	L	73	-	14.4	82.9	28.8	34.7
07	L	80	+2	9.1	72.1	24.2	33.2
08	L	65	-	11.6	85.5	31.5	36.4
09	P	47	+1	10.0	78.9	28.0	35.4
10	P	43	+2	9.6	80.3	28.5	35.5
11	P	72	+1	10.1	85.4	27.9	32.6
12	P	62	+1	12.0	81.9	28.0	34.2
13	L	78	+2	9.7	78.0	26.4	33.8
14	L	64	+1	13.5	90.1	32.0	35.5
15	L	62	-	10.6	85.3	29.2	34.2
16	L	24	+3	12.7	83.8	27.8	33.2
17	P	46	-	12.2	87.1	29.6	34.0
18	L	59	+1	11.5	91.1	32.0	35.2
19	P	49	-	13.2	86.9	29.1	33.5
20	P	62	+3	12.2	82.4	28.2	34.2
21	P	44	+1	10.0	83.2	28.2	33.9
22	P	43	+2	9.6	79.0	26.9	34.1
23	P	72	+1	10.6	91.6	32.1	35.1
24	P	47	+3	10.0	94.9	31.9	33.7
25	L	70	+3	10.7	84.9	28.4	33.4
26	L	60	+1	11.2	86.5	29.4	34.0
27	P	60	+2	11.4	80.5	27.4	34.0
28	L	49	+3	12.5	92.3	29.8	32.2
29	P	73	+3	8.2	81.8	28.2	34.5
30	P	18	+2	9.1	80.3	28.5	35.5
Average				10.9	83.3	28.5	34.24
Top Rated			+3	14.4	94.9	32.1	36.4
Lowest Value			-	8.2	71.1	23.8	32.2

**Table 4.2:** Characteristics of types of anemia by erythrocyte index

Normal		Anemia Normocytic Normochromic		Anemia Normocytic Hippodrome		Anemia Microcytic Normochromic	
N	%	n	%	n	%	n	%
6	20%	18	60%	3	10%	3	10%

**Table:** 

Correlations			
		Blood Urine	Hemoglobin

Blood Urine	Pearson Correlation	1	-.369^*^

	Sig. (2-tailed)		.045

	N	30	30

Hemoglobin	Pearson Correlation	-.369^*^	1

	Sig. (2-tailed)	.045	

	N	30	30
*. Correlation is significant at the 0.05 level (2-tailed).			

Table 4.1 shows the results of dipstick blood urine examination and erythrocyte index in UTI patients with average Hb, MCV, MCH, MCHC values respectively are 10.9 g / dL, 83.3 fl, 28.5 pg, and 34.24%. In the results of the blood urine examination, the highest positivity value was positive 3 (+3) and the lowest was negative.

**Figure 4.3:**
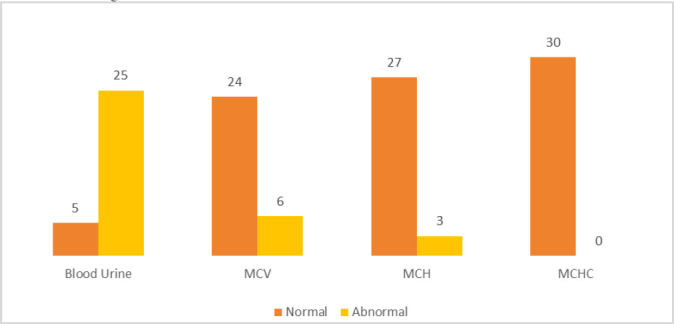
Number of criteria for blood urine test results and erythrocyte index in UTI patients

Based on Figure 4.3, the results of the examination on 4 parameters obtained abnormal results, namely a decrease in Hb levels in 25 samples (83%), MCV 6 samples (20%), MCH 3 samples (10%) and MCHC 0 samples (0%) from a total of 30 examination samples.

Based on the results of data analysis on blood urine dipstick method on the incidence of anemia in UTI patients, a significant result (p<0.05) was obtained which showed a correlation between blood urine and anemia, and based on the results of the Pearson correlation test obtained, a degree of -0.363 which showed a weak correlation. Blood urine is negatively related to the incidence of anemia (Hb levels), so the higher the positive value of blood urine, the lower the Hb value.

**Table 4.4:** Interpretation of the correlation coefficients of the person correlation test

Coefficient Interval	Relationship Level
0.000 – 0.199	Very Low
0.200 – 0.399	Low
0.400 – 0.599	Keep
0.600 – 0.799	Strong
0.800 – 1.000	Very Powerful

## Discussion

The study was conducted to determine the relationship between the positive level of blood urine dipstick method to the incidence of anemia in urinary tract infection (UTI) patients. The results showed that the sample of UTI patients in women was more than men, namely 18 women (60%) while men 12 people (40%) from a total of 30 study samples. This is in agreement with the report of Purnama *et al*. (2018) that urinary tract infection is one of the most common infections in women, accounting for about 25% of all infections that occur in women. About 50-60% of women will feel the symptoms of urinary tract infection during her lifetime. Based on the results of the study, it was found that most patients were women; as many as 26 people (65%), while men were just 14 people (35%). From the results of the study obtained, women are more at risk of developing urinary tract infections, because anatomically the length of the female urethra is shorter than men, the size of the urethra in women is less than 3-5 cm while in men it is 23-25 cm (Purnomo, 2011). Another study conducted by Suharjo *et al*. (2022) examined the incidence of UTIs in boys who are more susceptible to UTIs in the first year of life compared to girls. Therefore, in general, women dominate urinary tract infections. Based on the age of respondents, the sample of UTI patients with an age range over 50 years is more (16 people 53%), than the age of people of age less than 50 years, a total of 14 people (47%) from a total of 30 research samples. This is also in concordance with the results of the study by Sari and Muhartono (2018). The prevalence of UTIs in female DM patients is 43% and in male DM patients is 30% (Ravinder Singh *et al.*, 2011). Almost 50% of women have experienced symptoms of, or treatment of UTI once in their lifetime (Foxman, 2002). The prevalence of UTIs in female DM patients is 43% and in male DM patients it is 30 (Ravinder Singh *et al.*, 2011).

Examination of the erythrocyte index in UTI patients with mean Hb, MCV, MCH, MCHC values were 10.9 g/dL, 83.3 fl, 28.5 pg, and 34.24% respectively. The results of study I show values that are below normal, this is likely to occur as a result of the occurrence of bleeding in the urinary tract that is likely caused by urinary erythrocytes that exceed normal value (Syarif *et al.*, 2020) While in the results of blood urine examination, the highest positivity value was positive 3. This shows the condition of patients who experience UTIs and the results of this study are in line with the results of research conducted by Hasan (2021). Urinary tract infection patients who experience hematuria have a urinary erythrocyte count of >3 cells/LP. There were also 10 people (33%) with a urine erythrocyte count of 0-3 cells/LP. The results of this study are also in line with the results of research from(Fitria, Indah and Tjekyan, 2014), a results of high incidence of anemia in children suffering from UTIs and high prevalence of anemia in children suffering from urinary tract infections with positive hematuria.

UTI diagnosis done with complete urine examination includes macroscopic, chemical, and microscopic examinations. Chemical examination is done by dipping method (*dipstick*), whereas microscopic urine analysis is done by looking at the image of the sediment urine(Rinawati and Aulia, 2022). One of the parameters on the dipstick is blood. Examination with reagent strips (dipstick) serves to detect erythrocytes, free hemoglobin, and myoglobin (Hatman, Semedi and Budiono, 2021). So that the presence of erythrocytes, myoglobin and hemoglobin in the urine will give a positive result on the urine blood dipstick parameter which means hematuria occurs. Hematuria is an abnormal condition characterized by the presence of erythrocytes in the urine. Damage in the structure of the urinary tract causes hematuria so that blood will mix with urine in an abnormal state. Erythrocytes are normally absent in the urine, although their presence is 1–2 per large field of view is still considered as normal (Dwiyana and Astrawinata, 2016).

## Conclusions

Based on the research conducted, several things can be concluded as follows: 

a. The results of the dipstick blood urine examination obtained the highest positivity value of positive 3

b. Rerate values of Hb, MCV, MCH, MCHC sequentially are 10.9 g/dL, 83.3 fL, 28.5 am, and 34.24%.

c. The results of the pearson correlation test obtained a relationship degree of -0.363 which shows a weak correlation. Blood urine is negatively related to the incidence of anemia (Hb levels), so the higher the positive value of blood urine, the lower the Hb value.

### Conflict of Interest:

The authors declare that there is no conflict of interest associated with this study.

**Abbreviations:**
(UTI); Urinary Tract Infection:
(Hb); Hemoglobin:
(TBC); Tuberculosis:
(MCV); Mean Corpuscular Value:
(MCH); Mean Corpuscular Hemoglobin:
(MCHC); Mean Corpuscular Hemoglobin Concentration:

## References

[1] Andriani R (2019). 'Peranan Pencitraan dalam Deteksi Kelainan Anatomik pada Anak dengan Infeksi Saluran Kemih Atas'. Majalah Kedokteran FK UKI.

[2] Chaparro C. M, Suchdev P. S (2019). 'Anemia epidemiology, pathophysiology, and etiology in low-and middle-income countries'. Annals of the New York Academy of Sciences.

[3] Dwiyana Y, Astrawinata D. A (2016). 'Perubahan Bentuk Eritrosit Di Glomerulonefritis'. Indonesian Journal of Clinical Pathology and Medical Laboratory.

[4] Fitria B, Indah H, Tjekyan R (2014). 'Prevalensi Anemia pada Anak yang Menderita Infeksi Saluran Kemih'. Majalah Kedokteran Sriwijaya.

[5] Foxman B (2002). 'Epidemiology of urinary tract infections:incidence, morbidity, and economic costs'. The American Journal of Medicine.

[6] Handayani Wiwik, Haribowo A. S (2008). Asuhan Keperawatan pada Klien dengan Gangguan Sistem Hematologi.

[7] Hatman F. A, Semedi B. P, Budiono B (2021). 'Analisis Faktor Risiko terhadap Lama Perawatan Pasien Sepsis yang Meninggal di Ruang Perawatan Intensif RSUD Dr. Soetomo Surabaya'. JAI (Jurnal Anestesiologi Indonesia).

[8] Inayati I, Falah K (2014). 'Uji Diagnostik Urinalisis Lekosit Esterase terhadap Kultur Urin pada pasien Infeksi Saluran Kemih (ISK) dengan Kateterisasi Uretra'. Syifa'MEDIKA:Jurnal Kedokteran dan Kesehatan.

[9] Kurniawan S. P (2018). 'Sistem Pakar untuk Diagnosis Penyakit Anemia Menggunakan Metode Certainty Faktor dengan Mesin Inferensi Forward Chaining Berbasis Web'. Jurnal Mahasiswa Teknik Informatika.

[10] Purnomo B. B (2011). Dasar-Dasar Urologi.

[11] Ravinder Singh C, Nelson R, Muthu Krishnan P, Pargavi B (2011). 'Identification of volatile constituents from Premna serratifolia L. through GC-MS'. International Journal of PharmTech Research.

[12] Rinawati W, Aulia D (2022). 'Update in Laboratory Diagnosis of Urinary Tract Infection'. Jurnal Penyakit Dalam Indonesia.

[13] Riswanto;Rizki M (2015). Urinalisis:Menerjemahkan Pesan Klinis Urine.

[14] Sugianto, Magendha I. W, Suwiyoga K, Suwardewa T. G. A, Mayura I. G. P, Suardika A, Putra I. W. A (2020). 'Infeksi Saluran Kemih Sebagai Faktor Risiko Terjadinya Persalinan Preterm'. Intisari Sains Medis.

[15] Sugiyono (2017). Motode Penelitian Kuantitatif, Kualitatif, dan R&amp;D.

[16] Syarif J, Riskayanti (2020). 'Perbandingan Hasil Pemeriksaan Leukosit dan Eritrosit Urin Menggunakan Urin Pagi dan Sewaktu Metode Carik Celup pada Penderita Infeksi Saluran Kemih'. Jurnal Media Laboran.

[17] Vidiasari D, &amp; P (2016). 'Gambaran Karakteristik Ibu Hamil yang Mengalami Infeksi Saluran Kemih (ISK) di Wilayah Kerja Puskesmas Pekauman Banjarmasin'. Dinamika Kesehatan.

[18] Vincent J. L (2002). 'Anemia and Blood Transfusion in Critically Ill Patients'. The Journal of the American Medical.

[19] Zhou Y, Zhou Z, Zheng L, Gong Z, Li Y, Jin Y, Huag Y, Chi M (2023). 'Urinary Tract Infections Caused by Uropathogenic Escherichia coli:Mechanisms of Infection and Treatment Options'. International Journal of Molecular Sciences.

